# Neurology

**Published:** 1904-11-26

**Authors:** 


### NEUROLOGY.

**Babinski's Reflex.**—Goldflam[1] has made a study of Babinski's reflex in a large number of cases with the following results. When the sole of the foot is irritated two kinds of reflex are started—the cortical plantar and the spinal plantar. The former is the normal reflex, and consists chiefly in a flexion of the metatarso-phalangeal joints of the toes. If this path is impaired at any part of its course from the entry of the posterior root into the cord to the exit of the anterior root, or if the cortical centre is thrown out of action, as in normal sleep, this reflex is absent, and the stimulus passes by collaterals to the cells of the anterior horn — especially to the centre of the extensor longus hallucis, this constituting the spinal plantar reflex, or Babinski's, and there is extension of the great toe at the metatarso-phalangeal joint. The spinal plantar reflex is most usually caused by affections of the motor part of the cortical reflex path, because such are commoner than affections involving the whole of the sensory path. The presence of Babinski's reflex would therefore in most cases imply organic mischief in the brain or cord. The following summary of the more important points concerning the Babinski sign is given by Bramwell.[2] The patient should lie down on the side of the leg to be tested, with the thigh flexed on the trunk and the leg on the thigh, so that the muscles are relaxed. The observer grasps the dorsum of the foot with one hand, and with the other, in which he holds a quill or pin, stimulates the plantar surface, examining especially the outer part of the plantar surface and the ball of the great toe. When the feet are cold and damp, or after the patient has been standing some time, the plantar reflex is often absent. A single movement of extension, provided that it is undoubtedly reflex, is of the greatest significance. An extensor response is present in the great majority of infants, perhaps all. In healthy persons, after the first year of life, the plantar reflex is flexion. The Babinski sign (*i.e.* extension of the great toe at the metatarso-phalangeal joint) means implication of the upper motor neurons by organic disease. Exceptions to this statement are (*a*) the infantile response; (*b*) the extensor response, which takes place after an epileptic convulsion, (*c*) the extensor response which has been described in strychnine poisoning and in tetanus. Absence of the Babinski sign does not necessarily exclude organic disease of the upper motor neurons, for in some cases of old-standing hemiplegia the response is of the flexor type. The Babinski sign probably never occurs in a case of hysteria uncomplicated by organic disease.

**Epilepsy.**—Turner,[3] in a clinical lecture on the prognosis of epilepsy, said that a predisposition to epilepsy does not militate against a good result in an otherwise favourable case, but materially increases the probability of the disease becoming confirmed and the supervention of dementia. The age at which the disease commences is an important factor in prognosis. If fits commence under 10 years of age, considerably over 60 per cent. become confirmed epileptics; above that age until 30 the percentage which become confirmed steadily diminishes, from 30 to 35 it is large, whilst over 35 the outlook is relatively favourable. The longer the fits have lasted before treatment is begun the less favourable is the prognosis. The less frequent the fits the better is the prognosis. Cases of grand mal are easily influenced by treatment, those of petit mal only with difficulty. The majority of confirmed cases have both forms of seizure. Fits occurring only during sleep are less amenable to treatment than those occurring in waking hours. Marriage either produces no change at all or makes the fits worse, and if pregnancy take place the fits are particularly liable to occur at quickening, childbirth, and during lactation. If an absence of fits for eight years be taken as indicating a cure, then it may be said that 10 per cent. are cured. As regards treatment, it should be remembered that if bromides are going to be beneficial they will influence the disease in doses of from 30 to 45 grains per diem. If the case does not do well on that dose it probably will not do at all. The majority of cases should be given the medicine in one dose at bedtime. If there is any definite periodicity forestall the onset by a dose. If the fits are arrested for some months it is best to continue the bromide indefinitely in moderate doses.

**Uræmic Hemiplegia.**—It is well known that occasionally patients suffering from uræmia develop hemiplegia, lasting until death; but the nature and causation is still doubtful. Œdema of the brain substance and its membranes has been found in some cases, in others, however, it is absent, so that this cannot be the cause. The only experiments throwing any light on the problem are those of Raymond who removed the left superior cervical ganglion from a rabbit and several days later ligatured the ureter at the hilum in both kidneys, thus producing an artificial uræmia. The convulsions which occurred were limited to the right side of the body. In fatal cases of uræmia in man Ewing found marked change in the chromatic substance of the nerve cells, very irregular, however, in character and distribution. Weisenburg[4] has examined the central nervous system in two cases of uræmic hemiplegia, with the result that changes were found in the Betz cells of the paracentral lobule of both sides of the brain, though more intense on the side opposite to the paralysis. In addition there was a primary degeneration in the pyramidal fibres on the same side as the paralysis. No reason was discovered why the lesions were hemiplegic in distribution.

**Tuberculous Meningitis in Adults.**—Jackson,[5] from an analysis of 18 cases, finds that the symptoms of tuberculous meningitis in adults differ in many ways from those of the disease in children. In only three cases was there any history pointing to previous tubercular disease. On the other hand, in two cases only did the necropsy fail to reveal old tuberculous lesions, though in none was there extensive disease capable of diagnosis during life. The period of illness before admission was much shorter than in children. The prodromal symptoms were indefinite and suggestive of many acute fevers rather than of cerebral disease; and as a fact in most of the cases Widal's test was performed. One-third of the cases presented a state described as "typhoid delirium," and two-thirds had varying degrees of stupor and delirium. The kind of headache, cerebral cry, and restlessness usually present in children were absent. In only one case was headache severe. The pulse rate was slow at first, though later on rapid. The temperature varied from 100° to 101°. On lumbar puncture the cerebro-spinal fluid, which was under pressure, spirted out, almost clear, and containing a few flocculi, in only one case were tubercular bacilli found; it was thus very different from the semi-purulent fluid of acute cerebro-spinal meningitis in which organisms are easily obtained. In only one case was optic neuritis present. In 12 cases paralysis was limited to one or more ocular muscles; in two cases there was slight facial paralysis in addition. Other signs of cerebral disease, such as Kernig's sign, opisthotonus and retraction of the head, were less frequent and less marked than in cerebro-spinal meningitis. The course of the disease varied from one to three weeks.

[1] Dub. Med. Jour., July. [2] Scott. Med. and Surg. Jour., June.
[3] Clin. Jour., April 20. [4] Jour. of Nerv. and Ment. Dis., July.
[5] Boston Med. and Surg. Jour., May 12.

*(To be continued.)*

